# Utility of the narrow-band imaging international colorectal endoscopic classification for optical diagnosis of colorectal polyp histology in clinical practice: a retrospective study

**DOI:** 10.1186/s12876-021-01898-z

**Published:** 2021-08-28

**Authors:** Yasuhiko Hamada, Kyosuke Tanaka, Masaki Katsurahara, Noriyuki Horiki, Reiko Yamada, Tomomi Yamada, Yoshiyuki Takei

**Affiliations:** 1 Department of Gastroenterology and Hepatology, Mie University Hospital, 2-174 Edobashi, Tsu, Mie 514-8507 Japan; 2 Department of Endoscopy, Mie University Hospital, Tsu, Japan; 3 Department of Medical Innovation, Osaka University Hospital, Suita, Japan

**Keywords:** Colonoscopy, Colorectal polyp, Narrow-band imaging, NICE classification, Optical diagnosis

## Abstract

**Background:**

Narrow-band imaging (NBI) highlights the surface structures and vessels of colorectal polyps and is useful for determining the polyp histology. The narrow-band imaging international colorectal endoscopic (NICE) classification is a diagnostic tool for determining colorectal polyp histology based on NBI without optical magnification. In this study, we aimed to investigate the value of each type of the NICE classification for determining colorectal polyp histology using endoscopy data accumulated in a clinical setting.

**Methods:**

Endoscopy data for 534 colorectal polyps (316 patients) treated at our facility were retrospectively analyzed. First, we investigated the diagnostic performance of each type of the NICE classification for the optical diagnosis of colorectal polyp histology. The procedures were performed by experienced endoscopists using high-definition colonoscopy without optical magnification. Second, inter-observer and intra-observer agreements were assessed after providing experts and non-experts with a short lecture on the NICE classification. Using 50 fine NBI images of colorectal polyps without optical magnification, the inter-observer and intra-observer agreements between five experts and five non-experts were assessed.

**Results:**

The sensitivity, specificity, and accuracy values were 86.0%, 99.6%, and 98.5% for NICE type 1 lesions; 99.2%, 85.2%, and 97.8% for NICE type 2 lesions; and 81.8%, 99.6%, and 99.3% for NICE type 3 lesions, respectively. The inter-observer and intra-observer agreements ranged from substantial to excellent for both experts and non-experts.

**Conclusions:**

The NICE classification had good diagnostic ability in terms of determining the polyp histology and demonstrated a high level of reproducibility among experts and non-experts. Thus, the NICE classification is a useful clinical tool that can be used without optical magnification.

## Background

Colorectal cancer is one of the most frequently encountered malignancies and a common cause of cancer-related death in both men and women [1]. Colonoscopy with the removal of neoplastic polyps has been reported as an effective strategy for preventing deaths from colorectal cancer [2]. Colorectal polyps are routinely sent for pathological evaluation because pathological diagnosis is reliable for determining the appropriate interval until the next surveillance colonoscopy [3]. However, pathological evaluation via endoscopic biopsy or resection may cause adverse events, such as bleeding or perforation, and can incur high medical costs. If optical diagnosis via endoscopy can produce a diagnostic outcome equivalent to that of pathological evaluation, unnecessary removal of colorectal polyps may be avoided with significant reduction in medical costs.

Narrow-band imaging (NBI) is a diagnostic tool for visualizing the vessels and surface patterns of colorectal polyps. Developed in 1999, NBI has been reported to provide valuable information regarding the histology of polyps detected during colonoscopy [4]. Since then, many studies have reported the efficacy of NBI-assisted optical diagnosis of colorectal polyp histology [5–7]. Moreover, NBI-assisted optical diagnosis may enable immediate determination of the appropriate surveillance interval with reduction in the risk of adverse events and health care expenditure [8–10]. In Japan, several magnifying NBI classifications have been developed for use in clinical practice [11–14]. However, magnifying colonoscopy is not widely used globally [15, 16]. Therefore, there has been a need for a novel NBI classification that does not require optical magnification.

**Fig. 1:**
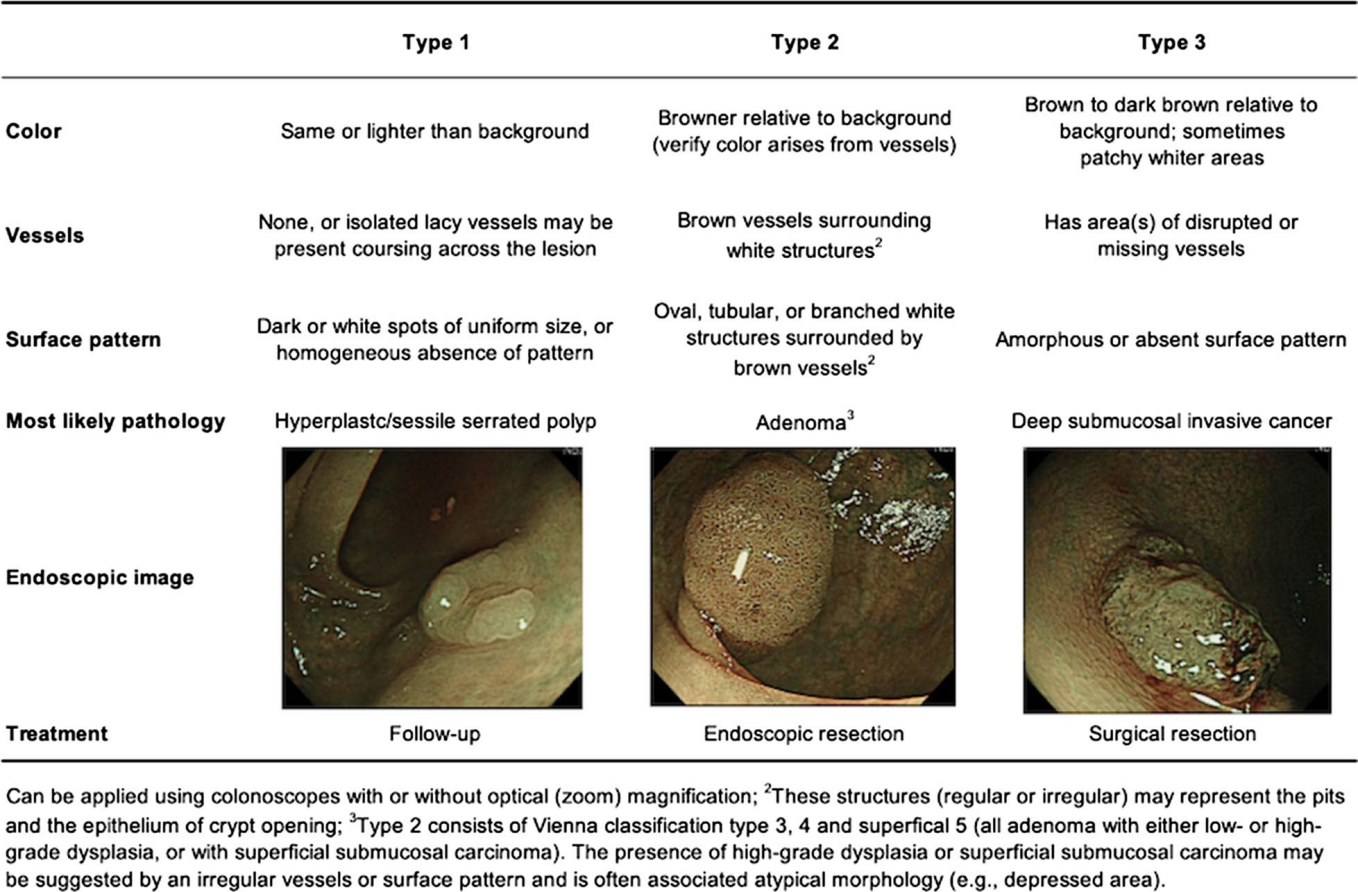
Narrow-band imaging International colorectal endoscopic classification

The Narrow-band imaging International Colorectal Endoscopic (NICE) classification, devised by the Colon Tumor NBI Interest Group, uses the color, vessels, and surface patterns of polyps to classify endoscopic findings without optical magnification (Fig. 1) [17–19]. This is the first NBI classification that can be used without optical magnification and is simplified for the ease of use. Previous studies have reported that the NICE classification is helpful for NBI-assisted optical diagnosis of colorectal polyp histology [6, 18, 20–28]. However, most of these studies were conducted to investigate the diagnostic outcomes of optical diagnosis for differentiating between neoplastic and non-neoplastic colorectal lesions. In contrast, there has been limited research on the diagnostic performance of each type of the NICE classification, and the findings were widely discrepant between the studies; the diagnostic accuracy of the NICE type 1, 2, and 3 was in the range of 36.5–92.6%, 80.0–90.7%, and 42.1–96.8%, respectively [16, 25, 29]. Thus, further analyses of data obtained in a clinical setting are required to determine the clinical value of each type of the NICE classification in NBI-assisted optical diagnosis of colorectal polyp histology.

This study aimed to determine the value of the NICE classification in NBI-assisted optical diagnosis of colorectal polyp histology by analyzing endoscopy data accumulated at our facility in a clinical setting. First, we evaluated the diagnostic performance of each type of the NICE classification for determining polyp histology, including the differentiation of neoplastic and non-neoplastic colorectal lesions. Second, we evaluated the reproducibility of the NICE classification for NBI images of colorectal polyps among both experts and non-experts.

## Methods

### Study design

**Fig. 2:**
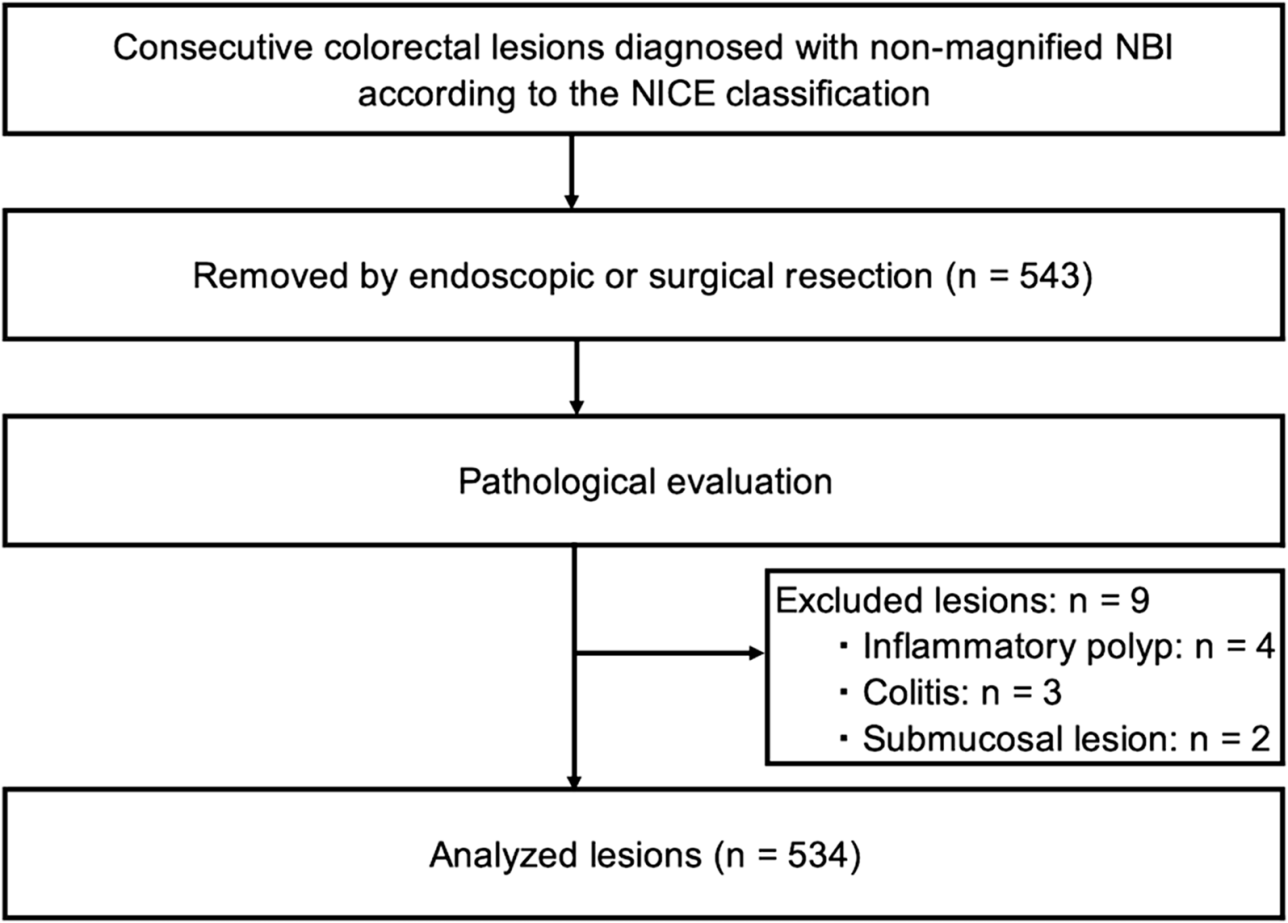
Study flow. *NBI* narrow-band imaging, *NICE classification* narrow-band imaging international colorectal endoscopic classification

This retrospective single-center study included 543 colorectal lesions (318 patients) with complete data, previously categorized using non-magnifying NBI observation according to the NICE classification. The details of these lesions were accumulated in the endoscopy database at our facility between April 2011 and March 2013. After the exclusion of nine lesions (two patients) that were pathologically diagnosed to be an inflammatory polyp, colitis, or a submucosal lesion, data for 534 colorectal lesions (316 patients) were available for the analysis (Fig. 2). The study was approved by the local ethics committee and conducted in accordance with the ethical standards of the Declaration of Helsinki. The ethics committee approved the use of an opt-out method of obtaining consent, and accordingly, informed consent was obtained via an opt-out option on the website of our facility.

### Diagnostic performance of the NICE classification for determining colorectal polyp histology

During the study period, all colonoscopies were performed by any of the six experienced endoscopists, each of whom had performed more than 3000 colonoscopies and had experience in using magnifying NBI for optical diagnosis but limited experience in using the NICE classification. Non-magnified NBI images were obtained using a high-definition colonoscope (CF-H260AZI, CF-Q260AI, PCF-Q260AZI, or PCF-Q260AI) combined with a standard video processor (EVIS LUCERA SPECTRUM; Olympus Medical Systems, Tokyo, Japan). After intubation of the cecum, the colonic mucosa was evaluated under white light during the withdrawal of the colonoscope. All polyps detected were documented for size, location, and morphology. Polyp size was estimated by comparison with the span of open biopsy forceps, sheath of the polypectomy snare, or diameter of an open snare placed against the polyp. Polyps were assigned to the cecum, ascending colon including the hepatic flexure, transverse colon including the splenic flexure, descending colon, sigmoid colon, or rectum. The Paris Classification System for Superficial Neoplastic Lesions in the Digestive Tract was used to define polyp morphology [30]. Each polyp identified under white light was further evaluated using NBI without optical magnification and immediately categorized as type 1, 2, or 3 according to the NICE classification. All polyps were removed using endoscopic forceps/snare or surgical resection, and the resected specimens were sent for pathological evaluation. The pathological diagnosis was made according to the World Health Organization criteria by two experienced pathologists. Based on the pathological characteristics, each lesion was identified as a hyperplastic polyp (HP), sessile serrated polyp (SSP), low-grade dysplasia (LGD), high-grade dysplasia (HGD; intramucosal cancer), superficial submucosal invasive carcinoma (SM-s; < 1000 µm of submucosal invasion), or deep submucosal invasive carcinoma (SM-d; ≥ 1000 µm of submucosal invasion).

### Inter-observer and intra-observer agreements by experts and non-experts

We assessed the inter-observer and intra-observer agreements among experts and non-experts to determine the validity and usefulness of this classification after providing a short lecture on the NICE classification. We prepared a set of fine typical NBI images of colorectal polyps of various NICE types for this evaluation. All images were required to be of sufficient clarity to be able to evaluate their color, vessels, and surface pattern and taken with color mode grade 3 and structure enhancement grade A8. After applying these criteria, non-magnified NBI images for 50 of the 534 lesions were selected for the agreement study. Of these 50 lesions, 10 were HP/SSP (type 1), 31 were LGD/HGD/SM-s (type 2), and 9 were SM-d (type 3). The NBI images of these lesions were arranged in random order. In accordance with the NICE classification, five experts who had performed > 3000 colonoscopies but had limited experience using the NICE classification and five non-experts (medical students) determined the type of NICE classification for the lesions identified using the NBI images twice, with a 2-week interval between the evaluations. All observers received a 30-min explanation outlining the concept of the NICE classification before the first evaluation. All observers were blinded to the pathological diagnosis and classification of the lesion. At the second evaluation, they were blinded to the results of their first evaluation.

### Statistical analysis

We evaluated the diagnostic performance (sensitivity, specificity, positive predictive value [PPV], negative predictive value [NPV], and accuracy) of each type of the NICE classification. Diagnostic performance was also assessed according to whether the polyp measured < 10 mm or ≥ 10 mm. Inter-observer and intra-observer agreements were analyzed using Cohen’s kappa statistic. Multiple comparisons of the kappa value between pairs of observers were conducted as a measure of the inter-observer agreement; 10 pairs could be made when a pair was chosen among five observers in each group, and they were compared. The kappa values for each group are presented as the mean, median, and range. The strength of agreement based on the kappa value was defined as follows: poor, ≤ 0.20; fair, 0.21–0.40; moderate, 0.41–0.60; substantial, 0.61–0.80; or excellent, 0.81–1.0. Fisher’s exact test was used to compare the diagnostic outcomes between polyps measuring < 10 mm and those measuring ≥ 10 mm. All statistical analyses were performed using SPSS version 24 (IBM Corp., Armonk, NY, USA) and EZR version 1.27 (Saitama Medical Center, Jichi Medical University, Japan) [31]. All statistical analyses were two-sided, and *P* values < 0.05 were considered statistically significant.

## Results

### Patients and polyp characteristics in the study cohort

The patient characteristics are summarized in Table 1. The median age was 69 (range 28–98) years. Of the total 316 patients included, 225 (71.2%) were male. The indication for colonoscopy was polyp surveillance in 77 patients (24.4%), endoscopic resection in 69 (21.8%), positivity of a fecal immunochemical test in 69 (21.8%), screening in 28 (8.9%), and positivity on positron emission tomography in 20 (6.3%).

**Table 1:** Patients characteristics of the study cohort

Patients, n	316
Median age, years (range)	69 (28–98)
Sex, male, n (%)	225 (71.2)
Indication for colonoscopy, n (%)	
Polyp surveillance	77 (24.4)
Endscopic resection	69 (21.8)
FIT positive	69 (21.8)
Screening	28 (8.9)
PET positive	20 (6.3)
Hematochezia	14 (4.4)
Anemia	12 (3.8)
Change of bowel habit	12 (3.8)
Tumor marker elevation	5 (1.6)
Others	10 (3.2)

*FIT* fecal immunochemical test, *PET* positron emission tomography

The polyp characteristics are summarized in Table 2. The median size was 6 (range 1–100) mm. In total, 237 lesions (44.4%) were in the right colon, 237 (44.4%) in the left colon, and 60 (11.2%) in the rectum. Pathological evaluation revealed that 491 (92.0%) were neoplastic polyps (LGD/HGD/SM-s/SM-d).

**Table 2:** Polyp characteristics of the study cohort

Lesions, n	534
Median size, mm (range)	6 (1–100)
Morphology n (%)	
Ip	36 (6.7)
Is, Is + IIc	424 (79.4)
IIa, IIa + IIc	71 (13.3)
IIc	3 (0.6)
Location, n (%)	
Right colon (cecum to transvese colon)	237 (44.4)
Left colon (descending colon to simoid colon)	237 (44.4)
Rectum	60 (11.2)
Pathological diagnosis, n (%)	
HP/SSP	43 (8.0)
LDG	427 (80.0)
HGD	48 (9.0)
SM-s	5 (0.9)
SM-d	11 (2.1)

*HP* hypeplastic polyp, *SSP* sessile serrated polyp, *LDG* low-grade dyplasia, *HGD* high-grade dysplasia, *SM-s* superfical submucosal invasive carcinoma (< 1000 mm), *SM-d* deep submucosal invasive carcinoma (≥ 1000 mm)

### Relationship between the NICE classification and polyp histology

The relationship between the NICE classification and polyp histology stratified by the polyp size is presented in Table 3. Independent of size, 39 lesions were diagnosed as NICE type 1 and 37 (94.8%) were HP/SSP; of 484 lesions diagnosed as NICE type 2, 476 (98.3%) were LDG/HDG/SM-s; and of 11 lesions diagnosed as NICE type 3 and 9 (81.8%) were SM-d.

**Table 3:**
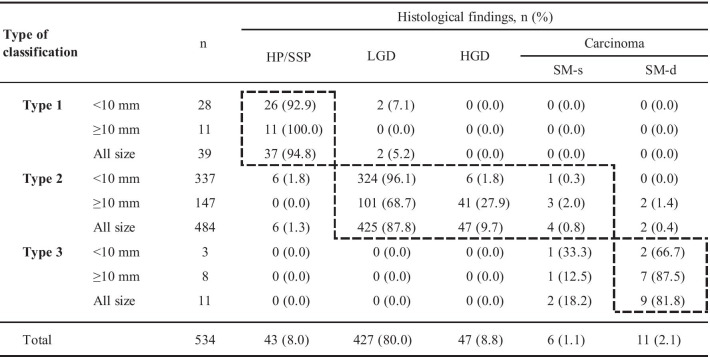
Relationship between each type in the NICE classification and polyp histology stratified by polyp size

Surrounded by dotted line: most like histology of each type in the NICE classification

*NICE classification* Narrow-band imaging international colorectal endoscopic classification, *HP* hypeplastic polyp, *SSP* sessile serrated polyp, *LDG* low-grade dyplasia, *HGD* high-grade dysplasia, *SM-s* superfical submucosal invasive carcinoma (< 1000 mm), *SM-d* deep submucosal invasive carcinoma (≥ 1000 mm)

### Diagnostic performance

The diagnostic performance for each NICE classification type when stratified by polyp size is presented in Table 4. The sensitivity, specificity, PPV, NPV, and accuracy were 86.0%, 99.6%, 94.9%, 98.8%, and 98.5% for NICE type 1; 99.2%, 85.2%, 98.3%, 92.0%, and 97.8% for NICE type 2; and 81.8%, 99.6%, 81.8%, 99.6%, and 99.3% for NICE type 3, respectively. Neoplastic lesions (NICE type 2/3) could be determined with a sensitivity, specificity, PPV, NPV, and accuracy of 99.6%, 86.0%, 98.8%, 94.9%, and 98.5%, respectively. After stratification by polyp size, the sensitivity, specificity, PPV, NPV, and accuracy of diagnosis of each type did not differ significantly according to whether the polyp measured < 10 mm or ≥ 10 mm.

**Table 4:** Diagnostic performance of each type in the NICE clasification stratified by polyp size

Type of classification	Sensitivity (%) (95% CI)	Specificity (%) (95% CI)	PPV (%) (95% CI)	NPV (%) (95% CI)	Accuracy (%) (95% CI)
Type 1					
< 10 mm	81.2 (63.6–92.8)	99.4 (97.9–99.9)	92.9 (76.5–99.1)	98.2 (96.2–99.3)	97.8 (95.8–99.1)
≥ 10 mm	100.0 (61.5–100.0)	100.0 (96.5–100.0)	100.0 (61.5–100.0)	100.0 (96.5–100.0)	100.0 (96.7–100.0)
All size	86.0 (72.1–94.7)	99.6 (98.5–100.0)	94.9 (82.7–99.4)	98.8 (97.4–99.6)	98.5 (97.1–99.4)
Type 2					
< 10 mm	99.1 (97.4–99.8)	82.4 (65.5–93.2)	98.2 (96.2–99.3)	90.3 (74.2–98.0)	97.6 (95.4–98.9)
≥ 10 mm	99.3 (96.2–100.0)	90.0 (68.3–98.8)	98.6 (95.2–99.8)	94.7 (74.0–99.9)	98.2 (94.8–99.6)
All size	99.2 (97.9–99.8)	85.2 (72.9–93.4)	98.3 (96.8–99.3)	92.0 (80.8–97.8)	97.8 (96.1–98.8)
Type 3					
< 10 mm	100.0 (9.4–100.0)	99.7 (98.5–100.0)	66.7 (9.4–99.2)	100.0 (98.5–100.0)	99.7 (98.5–100.0)
≥ 10 mm	77.8 (40.0–97.2)	99.4 (96.5–100.0)	87.5 (47.3–99.7)	98.7 (95.5–99.8)	98.2 (94.8–99.6)
All size	81.8 (48.2–97.7)	99.6 (98.6–100.0)	81.8 (48.2–97.7)	99.6 (98.6–100.0)	99.3 (98.1–99.8)
Determination of neoplastic polyp (Type 2/3)					
< 10 mm	99.4 (97.9–99.9)	81.2 (63.6–92.8)	98.2 (96.2–99.3)	92.9 (76.5–99.1)	97.8 (95.8–99.1)
≥ 10 mm	100.0 (96.5–100.0)	100.0 (61.5–100.0)	100.0 96.5–100.0)	100.0 (61.5–100.0)	100.0 (96.7–100.0)
All size	99.6 (98.5–100.0)	86.0 (72.1–94.7)	98.8 (97.4–99.6)	94.9 (82.7–99.4)	98.5 (97.1–99.4)

The sensitivity, specificity, PPV, NPV, and accuracy of each type do not differ significantly between polyp sized < 10 mm and ≥ 10 mm

*NICE classification* narrow-band imaging international colorectal endoscopic classification, *CI* confidence interval, *PPV* positive predictive value, *NPV* negative predictive value

### Inter-observer and intra-observer agreements

Inter-observer and intra-observer agreements were assessed using Cohen’s kappa values (Table 5). At the first reading, the average and median kappa values for inter-observer agreement were 0.86 and 0.86 (range 0.76–0.93) for experts and 0.59 and 0.62 (range 0.47–0.63) for non-experts, respectively, and those for intra-observer agreement were 0.82 and 0.83 (range 0.68–0.90) for experts and 0.63 and 0.63 (range 0.54–0.70) for non-experts, respectively.

**Table 5:** Inter-observer and intra-observer agreements of experts and non-experts for the NICE classification

	Kappa value		
	Average	Median	Range
Inter-observer agreement			
Experts	0.86	0.86	0.76–0.93
Non-experts	0.59	0.62	0.47–0.63
Intra-observer agreement			
Experts	0.82	0.83	0.68–0.90
Non-experts	0.63	0.63	0.54–0.70

Observers were experienced with > 3000 colonoscopies

Observers were medical students

*NICE classification* Narrow-band imaging international colorectal endoscopic classification

## Discussion

Two major findings of this study were as follows: First, this study demonstrated a good diagnostic outcome of each type of the NICE classification for determining polyp histology by evaluating the endoscopy data accumulated in a clinical setting. The sensitivity, specificity, PPV, NPV, and accuracy for diagnosing each NICE type did not differ significantly according to the polyp size. Second, there were good inter-observer and intra-observer agreements between non-experts as well as experts, confirming the reproducibility of the NICE classification.

The NICE classification had a specificity, NPV, and accuracy of > 90% for the diagnosis of type 1 and 3 lesions. These findings indicate that NICE types 1 and 3 are definite indicators of the most likely histology with significant diagnostic outcomes. Therefore, the NICE classification is useful for identifying polyps that do not need to be removed and those that warrant surgical resection [18, 19, 28]. For a diagnosis of NICE type 2, the NPV and accuracy were > 90%, but the specificity was 85.2%. This may be because NICE type 2 lesions show various pathological features, ranging from LGD to SM-s. However, optical diagnosis is important for differentiation between LGD/HGD/SM-s and SM-d lesions in clinical practice to determine the appropriate therapeutic strategy. The NICE classification will allow us to distinguish between LGD/HGD/SM-s lesions, which can be treated curatively via endoscopic resection, and SM-d lesions, which require surgical resection. Furthermore, the specificity, NPV, and accuracy for the diagnosis of NICE type 2/3 were 86.0%. 94.9% and 98.5%, respectively, which indicates that the NICE classification has good diagnostic performance for determining neoplastic polyps. Based on these results, the NICE classification can be considered a useful tool for determining the most likely histology of colorectal polyps in clinical practice.

These results are mostly superior to those of previous studies that investigated the application of each type of the NICE classification [16, 25, 29]. There are two likely reasons for the differences in the findings between these studies and our study. First, the diagnostic outcomes using the NICE classification were investigated based on examination by endoscopists skilled in NBI-assisted optical diagnostic systems. Although these endoscopists had limited experience in using the NICE classification before participating in this study, they had an experience of using magnifying NBI routinely, such as the Hiroshima classification [11] or Sano classification [12]. The NICE classification is similar to these classifications regarding the diagnostic strategy using the color, vessels, or surface patterns of colorectal polyps. Thus, the endoscopists might also be skilled in the NBI-assisted diagnostic strategy of the NICE classification. Wang et al. have reported that a group of highly experienced endoscopists who had routinely used magnifying NBI for > 5 years showed excellent diagnostic outcomes in NBI-assisted optical diagnosis using the NICE classification; the sensitivity, specificity, and accuracy were as follows: 84.6%, 94.9%, and 93.9% in type 1, 91.4%, 86.3%, and 90.7% in type 2, 91.7%, 97.0%, and 96.8% in type 3, respectively [16]. Second, the characteristics of the lesions may have influenced the diagnostic outcomes of our study. The percentages of HP/SSP (8.0%) and SM-d (2.1%) lesions in the present study were small. The data used had been obtained in a clinical setting; therefore, type 1 lesions in the rectosigmoid colon diagnosed as HP were not removed, and SM-d lesions are rarely encountered during routine colonoscopy in clinical practice. Consequently, almost all lesions were classified as NICE 2 type, contributing to this study’s diagnostic outcomes.

To ensure the generalizability of the NICE classification, we assessed the inter-observer and intra-observer agreements using 50 NBI images without optical magnification after providing experts and non-experts a lecture demonstrating the use of this classification. The global acceptance of NBI-assisted optical diagnosis in clinical practice will require a simple, standardized NBI classification that can be easily understood and applied by non-experts and experts. The NICE classification was developed with the guiding principles of simplicity and ease of use in mind. In the present study, good agreement was demonstrated among both non-experts and experts. Therefore, the NICE classification can be accepted globally and used by endoscopists with different experience level.

Several studies have reported that NBI with optical magnification enables endoscopists to accurately determine colorectal polyp histology. A meta-analysis of 27 studies on NBI magnification reported that the pooled sensitivity, specificity, PPV, and NPV for predicting neoplastic polyps were 95.8%, 85.8%, 92.9%, and 91.5%, respectively [32]. These values are not significantly different from those obtained in our study. Furthermore, the use of optical zoom magnifying colonoscopes is not yet widespread globally in clinical practice [15, 16]. Therefore, using the NICE classification without optical magnification might be more convenient and practical for determining colorectal polyp histology [23].

There remains an unanswered question in this study. A recent study has highlighted NBI features that may be used to distinguish SSP from HP; the characteristic NBI findings for SSP include the red cap sign, a cloud-like surface, dilated and branched vessels, and expanded crypt openings [33]. When combined with these findings, the NICE classification may improve our ability to diagnose SSPs. However, the present study was performed using data obtained before these characteristic findings of SSP were recognized, and endoscopists were not asked to classify type 1 lesions as HP or SSP. Therefore, it is not possible to conclude the diagnostic performance of the NICE classification for SSP based on our current findings.

This study has some limitations. First, it had a single-center design. Second, it did not include a control group, such as NBI or chromoendoscopy with optical magnification. Therefore, to provide solid evidence for the value of the NICE classification, studies that include a control group and a larger number of individuals are required. Third, the confidence level of optical diagnosis could not be assessed because the data analyzed were obtained retrospectively from information accumulated in clinical practice. Fourth, we did not remove all type 1 lesions during colonoscopy, which may have introduced a degree of sampling bias. The data analyzed were obtained in a clinical setting, and type 1 lesions diagnosed as HP in the rectosigmoid colon were not removed. Our study focused on the resected lesions, and unresectable lesions could not be evaluated. Therefore, it is likely that sampling biases resulted in a low percentage of type 1 lesions, thereby influencing the results of this study. Finally, the data used in this study were accumulated between 2011 and 2013, and more recent data could not be used. However, all procedures were performed using a high-definition colonoscope combined with a standard video processor, similar to the equipment used in more recent studies. Therefore, the diagnostic outcomes of the NICE classification documented in this study can be considered to be almost equivalent to those reported in any investigations using more recent data.

## Conclusions

This study found that the use of the NICE classification provided good diagnostic outcomes when determining colorectal polyp histology in a clinical setting, regardless of the polyp size. Furthermore, it demonstrated favorable reproducibility by non-experts as well as by experts. The NICE classification is practical for use without magnifying colonoscopy because it is simple and easy to apply and has high accuracy for diagnosis and therapeutic decision-making.

## Data Availability

All data generated and analyzed during this study are included in this published article.

## Abbreviations

NBI: Narrow-band imaging
NICE: Narrow-band imaging international colorectal endoscopic
HP: Hyperplastic polyp
SSP: Sessile serrated polyp
LGD: Low-grade dysplasia
HGD: High-grade dysplasia
SM-s: Superficial submucosal invasive carcinoma
SM-d: Deep submucosal invasive carcinoma
PPV: Positive predictive value
NPV: Negative predictive value
